# Understanding anhedonia: a qualitative study exploring loss of interest and pleasure in adolescent depression

**DOI:** 10.1007/s00787-019-01364-y

**Published:** 2019-07-03

**Authors:** Rebecca Watson, Kate Harvey, Ciara McCabe, Shirley Reynolds

**Affiliations:** School of Psychology and Clinical Language Sciences, University of Reading, Reading, UK

**Keywords:** Anhedonia, Depression, Adolescence, Qualitative, Understanding

## Abstract

Anhedonia (or loss of interest and pleasure) is a core symptom of depression and may predict poor treatment outcome. However, little is known about the subjective experience of anhedonia, and it is rarely targeted in psychological treatment for depression. The aim of this study is to examine how young people experience anhedonia in the context of depression. Semi-structured interviews were conducted with 34 adolescents with a primary diagnosis of depression (*N* = 12) or elevated depressive symptoms (*N* = 22). Thematic analysis was used to identify important aspects of adolescents’ experiences. Four main themes were identified: (1) experiencing a loss of joy and a flattening of emotion; (2) struggling with motivation and active engagement; (3) losing a sense of connection and belonging; and (4) questioning sense of self, purpose, and the bigger picture. The results challenge the framing of anhedonia as simply the loss of interest and pleasure. Adolescents reported a range of experiences that mapped closely onto the cluster of negative symptoms associated with schizophrenia and were similar to the sense of ‘apathy’ characteristic in Parkinson’s disease. This highlights the potential benefit of taking a trans-diagnostic approach to understanding and treating reward deficits associated with mental health problems.

**Electronic supplementary material:**

The online version of this article (10.1007/s00787-019-01364-y) contains supplementary material, which is available to authorized users.

## Introduction

Anhedonia is a core symptom of major depressive disorder (MDD). It is defined by the DSM-5 as ‘markedly diminished interest or pleasure in all, or almost all activities most of the day, nearly every day’ [1]. The first onset of MDD frequently occurs in adolescence, with up to 20% of young people experiencing a depressive episode by the age of 18 [2]. In adolescents, depressed mood/irritability or anhedonia must be present for a depression diagnosis [1]. Between 50 and 78% of young people with a diagnosis of MDD report anhedonia in the UK [3, 4]. Anhedonia has been identified as a potential predictor of poor treatment outcome in adolescents above and beyond all other depression symptoms [5]. It may also be key to understanding suicidality, with adolescent suicide attempters reporting greater anhedonia severity than suicide ideators, even after controlling for depression and anxiety [6]. Despite the importance of anhedonia for diagnosis and prognosis, a variety of conceptual and methodological challenges exist meaning that the symptom of anhedonia is not well understood.

Research using functional MRI and behavioural tasks suggest that there are distinct components of anhedonia related to liking (consummatory/hedonic impact), wanting (anticipatory/motivation) and learning (reward prediction) [7, 8]. Although behavioural studies report no deficits in consummatory anhedonia in adults [9, 10], neural differences have been found during consummation in adults at risk of depression and adolescents with depression symptoms [11–13]. A deficit in reward anticipation is consistently reported in depressed adults and adolescents both at the neural [14] and behavioural level [15], whilst studies examining reward learning also find depressed adults have a reduced ability to behaviourally learn about reward [16, 17]. Taken together, this suggests that the concept of anhedonia may be better defined as a deficit in multiple aspects of reward processing.

The clinical assessment of anhedonia in young people is based on either administration of semi-structured diagnostic interviews, of which the Kiddie-Schedule for Affective Disorders and Schizophrenia (K-SADS) [18] is the gold standard, or on self-report questionnaires (e.g. Snaith Hamilton Pleasure Scale; SHAPS) [19]. Both methods rely on young people giving an accurate description of the symptom and its components. Most questionnaire measures have been developed for and with adults and contain items that are of doubtful relevance to young people. For example “I would enjoy a cup of tea, coffee, or my favourite drink” (SHAPS) [19] and “The sound of crackling wood in the fireplace is relaxing” (Temporal Experience of Pleasure Scale; TEPS) [20]. The most recent self-report questionnaires aim to assess components of anhedonia, e.g. consummatory (liking) and anticipatory (wanting) anhedonia. However, factor analysis shows that participants’ responses load on to separate factors that reflect rewards from different types of activities, namely general versus intimate aspects of social pleasure (i.e. Anticipatory and Consummatory Interpersonal Pleasure Scale; ACIPS) [21, 22], or hobbies versus sensory pleasures (i.e. Dimensional Anhedonia Rating Scale; DARS) [23]. The TEPS [20] is the only questionnaire in which separate factors for ‘liking’ and ‘wanting’ emerge, but these are highly correlated.

These limitations mean that it is unclear how best to assess adolescent anhedonia. Qualitative studies are needed as we do not know the best way to capture this experience in adolescents. Previous qualitative studies [24, 25] have explored aspects of adolescents’ experiences of depression but none have specifically elicited adolescents’ experiences of anhedonia in the context of depression. Therefore, the aim of this study was to explore how adolescents with a depressive disorder (or elevated symptoms of depression) experience anhedonia. One to one qualitative interviews allowed this topic to be explored sensitively. Thematic analysis ensured an in-depth exploration of the data, whilst enabling the research to capture a breadth and diversity of experiences.

## Methods

Ethical approval for the study was obtained from the University of Reading Research Ethics Committee and NHS Research Ethics Committee. Guidelines for ensuring rigour and reflexivity in qualitative research were followed [26], as well as the COREQ checklist for reporting qualitative data [27].

### Participants and recruitment

Participants included adolescents aged 13–18 years recruited from the community (age, *M* = 15.4, SD 1.6; gender, 55% male) or a clinical service (age, *M* = 15.6, SD 1.5; gender, 42% male).

Community participants were recruited through their school. Eighteen schools in the South of England were approached and 3 agreed to take part in the study (2 co-educational and 1 single-sex school). A single-sex school enabled male participants to be well represented within the study, as male participants are typically under-represented in clinical samples [3]. Based on the index of free school meals, the 3 schools differed on socio-economic status (proportion of children eligible for free school meals was 2%, 10% and 15%; 12% is the UK average) [28]. We screened 715 adolescents (approximately 40% of those invited to take part) for symptoms of depression using the Mood and Feelings Questionnaire (MFQ: long version) [29]; or the Short Mood and Feelings Questionnaire (SMFQ, short version) [30]. MFQ data from two of the three schools was collected in collaboration with other researchers for ongoing projects. The MFQ is the recommended screening tool for depression in the UK [31] and has good reliability and moderate diagnostic accuracy [32]. From this sample, we purposively sampled 30 young people seeking diversity of age, gender and a range of MFQ scores above the clinical cutoff to capture the breadth of depression severity (minimum scores for inclusion 27 on the MFQ [32] and 8 on the SFMQ [33]). Adolescents identified were invited to take part in this study approximately 2 weeks after completing the questionnaire. Twenty-two (73%) of those invited participated in the study, and 8 (27%) did not respond to requests to participate.

Clinical participants were recruited from referrals to a Child and Adolescent Mental Health Service (UK) in the South of England. As part of the routine clinical assessment young people, completed two semi-structured diagnostic interviews; the Kiddie-Schedule for Affective Disorders and Schizophrenia depression section and psychosis screen (K-SADS-L) [18] and the Anxiety Disorders Interview Schedule for Children (ADIS-IV-C/P) [34]. Twenty young people who met DSM-5 criteria for a primary diagnosis of depression [1] were invited to take part in the research. Twelve (60%) participants gave consent or assent to take part; and 8 (40%) declined or did not respond to requests to participate. Of those who took part, 11 met criteria for Major Depressive Disorder and one for Persistent Depressive Disorder.

### Procedure

A topic guide was developed using the authors’ clinical experience and research expertise in the fields of depression, anhedonia and qualitative methodology. Questions were evaluated by clinical experts, piloted on adolescents, and revised accordingly. The topic guide explored the following: (1) current and past interests and hobbies; (2) future enjoyment and plans; (3) changes and/or loss of enjoyment and interest. The topic guide was used flexibly and comprised open questions relating to pleasure and enjoyment, followed by prompts to gather richer data about each experience.

Informed written consent was obtained from all participants, and from the parents of young people under 16 years of age. The first author (RW), a female Ph.D. student, conducted all the interviews face-to-face. They took place in a quiet room at the school or clinic with only the researcher and participant present. Interviews were audio recorded and lasted an average of 33 min (range 17–73 min). Participants received a £10 gift voucher for their participation. Theoretical saturation was reached, with the data collection process no longer offering any new or relevant insights. Interviews were transcribed verbatim by RW, and all identifying information removed and pseudonyms assigned. Field notes were made after the interview and Nvivo software used to aid in analysis.

### Analysis

Thematic analysis (TA) was used to identify and analyse patterns of meaning in the dataset, highlighting the most salient clusters of content. This method is best suited for exploring a group’s conceptualisation of a specific phenomenon [26]. TA is not connected to a specific ontological or epistemological position; therefore, in this study, the researchers adopted a broadly critical realist (post-positivist) perspective [35]. This position makes the assumption that reality is measurable and observable, whilst acknowledging that participants are not fully aware of all the factors that influence their experiences [26]. The researchers considered their own sources of bias and prior assumptions, including knowledge and experience gained from working in child and adolescent mental health services (RW, SR) and conducting research into young people’s mental health (KH, CMcC, SR).

Constant comparative techniques were used to analyse the data, based on Braun and Clark’s [36] six stage thematic analysis method. In stage (1), the first author became familiar with the data by conducting and transcribing the interviews, and then reading and re-reading the transcripts. In stage (2), RW conducted line by line coding. Coding was an inductive and recursive process, with constant comparisons made between and within transcripts. All data were initially coded for both explicit and implicit meaning. Only information regarding unique personal circumstances, or treatment was categorised as ‘wider content’. The labelling of codes focused on capturing the experience of anhedonia. In stage (3), codes were combined into potential themes, which reflected major features and patterns in the data. In stages (4) and (5), themes were reviewed by examining all codes and themes collectively. As recommended by Saldana [37], tentative themes were reviewed by the research team (RW, KH, CMcC and SR). During these coding meetings, alternative interpretations were considered and discussed until a consensus on the interpretation of patterns in the data was reached. In the last stage, stage (6), agreed themes were finalised and quotations illustrative of each theme were identified.

## Results

See Table 1.

**Table 1:** Participant demographics and clinical characteristics

Pseudonyms	Age^a^	Gender	Ethnicity	MFQ		SHAPS score (/56)	Sub-sample
				Long score (/66)^b^	Short score (/26)^b^		
Adam	17	Male	White British	18	–	39	Clinical
Alice	13	Female	White British	37	–	32	Clinical
Amy	15	Female	White British	–	24	40	Community
Anna	13	Female	White British	–	10	46	Community
Ben	14	Male	Other	31	–	37	Community
Carl	16	Male	White British	59	–	37	Community
Chris	15	Male	White British	–	11	46	Community
Claire	17	Female	White British	56	–	30	Clinical
Elliot	16	Male	Other Asian background	30	–	29	Clinical
Gary	16	Male	White British	46	–	33	Clinical
Helen	17	Female	White British	–	13	50	Community
Ivy	13	Female	White British	39	–	35	Clinical
Isla	15	Female	Other Asian background	–	15	36	Community
India	16	Female	White British	46	–	35	Clinical
Jacob	16	Male	White British	45	–	26	Clinical
Jasmine	14	Female	White British	26	–	23	Clinical
Jayden	15	Male	White British	41	–	36	Clinical
Jennifer	17	Female	White British	42	–	37	Clinical
Joanne	15	Female	White British	–	16	38	Community
Karly	14	Female	White British	–	22	16	Community
Lucy	16	Female	White British	44	–	34	Clinical
Maya	15	Female	White British	–	21	41	Community
Mel	13	Female	Pakistani or Pakistani British		10	47	Community
Matthew	18	Male	White British	32	–	53	Community
Maddie	15	Female	White British	–	18	46	Community
Neil	15	Male	Chinese	33	–	44	Community
Quentin	15	Male	Other mixed background	–	11	43	Community
Richard	18	Male	Other White background	27	–	40	Community
Ross	18	Male	White British	37	–	32	Community
Stuart	16	Male	Other White background	31	–	44	Community
Tessa	17	Female	White British	–	20	**–**	Community
Tylor	15	Male	Other White background	31	–	39	Community
Theo	13	Male	White British	27	–	43	Community
Timothy	17	Male	White British	34	–	40	Community

*MFQ* Mood and Feelings Questionnaire (higher scores indicate more depression). Participants completed either the long or short MFQ. *SHAPS* Snaith Hamilton Pleasure Scale (higher scores indicate more pleasure)

^**a**^Age at interview

^**b**^MFQ score at screening or diagnosis

### Overview of themes

**Fig. 1:**
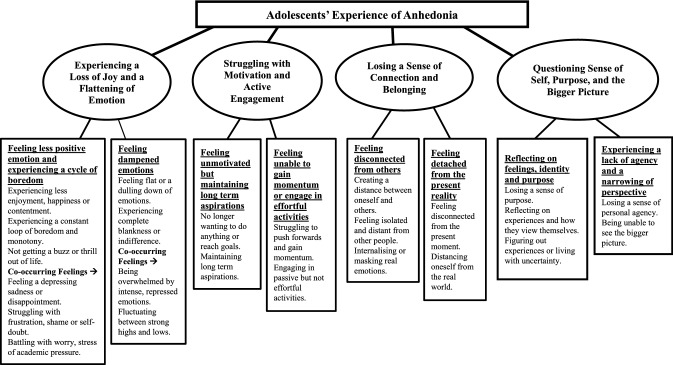
Diagram showing the interview topic (level 1), themes (level 2), sub-themes (level 3), and higher level codes (level 4)

Adolescents’ experiences were captured in four main themes: (1) experiencing a loss of joy and a flattening of emotion; (2) struggling with motivation and active engagement; (3) losing a sense of connection and belonging; (4) questioning sense of self, purpose, and the bigger picture (see Fig. 1). Each theme highlighted a unique aspect of adolescents’ experiences; however, there were areas of conceptual overlap. All major themes and sub-themes were expressed by both the clinical and community sub-samples.

**Theme 1—Experiencing a loss of joy and a flattening of emotion:** “I lost what I enjoyed doing” (Tessa, community)

This theme concerns the disruption of adolescents’ positive emotions and co-occurring negative emotions.

*Sub-theme: Feeling less positive emotion and experiencing a cycle of boredom*

Young people used a range of words to describe the presence or absence of positive emotions, including “enjoyment”, “interest”, “satisfaction”, “pride”, “curiosity”, “fun”, “endorphin rush”, “excitement”, “enthusiasm”, “relaxing”, “good” and “happy.” They described positive emotions as arising from being creative, active, achieving something difficult and spending time with others. A number of adolescents recruited from the clinic and the community described feeling a global loss of interest and enjoyment in anything. They described everything (or almost everything) as “boring”. This often seemed to contradict initial descriptions of hobbies and interests they took part in, or for which they expressed a liking or preference. These feelings of boredom and disinterest were closely linked with not wanting to do things. Adolescents also felt a sense of “monotony”, and described feeling like they were in a “constant loop” of mundane activities.

“I was just like completely bored with it. Like you get bored with a TV show, and you're like, okay leave it then, just move onto another one. It's quite like that with reading and sport because it was just a, I'm bored with this, let's try and find another thing and then I never really found another thing, I just try and do a lot of different things, think I was interested in them then get bored, and just get into a cycle of boredom, finding something else, bored with that, move on” (India, clinic).

Concepts such as “excitement” and “enthusiasm” were used by some participants in reference to both current enjoyment and the anticipation of future events. For example, Jayden (clinic) said “I get really excited watching TV, ‘cos I get really into what I’m watching”. For others excitement was absent, e.g. “nothing feels exciting” (Ivy, clinic), or things “sap the enthusiasm out of me” (Stuart, community). Many young people could not think of anything they were looking forward to or excited about, however, it was often unclear whether this was because they did not anticipate having fun, could not imagine future events, or had a “bad memory” for remembering what they had planned (i.e. prospective memory problems).

“If there's something new and shiny, new film or game I’ll probably be like, yeah it will come out and I’ll see it, but most stuff doesn’t get me excited, just sort of, wait for it to turn up and see how to feel about it.” (Ross, community).

While adolescents typically described a change in positive emotions; some also described feelings of sadness, anxiety and shame. These negative emotions sometimes resulted in a loss of positive feelings.

*Sub-theme: Feeling dampened emotions*

When asked to describe their feelings, some adolescents reported a partial or complete blunting of any emotion. They described themselves as feeling “dull”, “grey”, “flat”, “vacant”, “a blank sheet”, “empty” and “emotionless”. Some young people described a general flatness, passivity, and feeling “indifferent” or not “caring” about anything. Jacob (clinic) described special events like his birthday as feeling like “just another day”. This flatness was also displayed vocally by them talking with little intonation. Most young people’s experiences reflected a loss of both positive and negative emotion.

“I didn’t really feel anything, like, there was no like happiness or excitement, but there was also like no sadness. It was just like everything was grey.” (Carl, community).

The sense of blunted emotions was not reported all the time or by all young people. Sometimes adolescents felt strong fluctuations in mood, with their feelings shifting abruptly from happiness to sadness.

**Theme 2—Struggling with motivation and active engagement:** “I’m never motivated to do anything” (Jasmine, clinic)

This theme captures changes in wanting to do things, effort exerted and types of activities young people engaged in.

*Sub-theme: Feeling unmotivated but maintaining long term aspirations*

Adolescents described changes in how much they “wanted” or felt “motivated” to act or engage in experiences. This lack of drive often contrasted with young people’s stated long-term ambitions and goals, for example going to university, or playing in a band. Some participants’ lack of drive was related to specific experiences such as going to school or seeing friends. Others described a more global lack of drive, with them “not wanting to do anything at all” or even “not wanting to live” (Amy, community).

“Yeah like although they were the things I enjoyed, although I knew I should be enjoying them, for some reason like, I just like wouldn't have the motivation to do it.” (Ivy, clinic).

A loss of drive often occurred alongside a lack of positive emotion. Some participants, however, reported that although they had no drive at all, when they engaged in activities they did enjoy them. This was reported by young people in both the clinical and community samples.

*Sub-theme: Feeling unable to gain momentum or engage in effortful activities*

Many adolescents were less willing to make efforts needed to reach their goals or felt that everything required more effort. Young people often said “I just can’t be bothered” or that they had to “force” themselves to do anything. Many participants noted that making an effort to get things done was the key barrier to engaging with life and to improving their mood. Jennifer (clinic) said “it’s like if you have a wheel, starting to push the wheel is a lot of effort but the momentum will carry it forwards”. Young people sometimes linked their lack of mental and physical effort to low levels of energy and fatigue as well as to a lack of drive. When feeling unmotivated and fatigued, a number of adolescents said they did very little, often spending their free time just lying in bed.

“Most of the time it's my parents forcing me to get out of bed, other times if I actually have something planned, just sort of, force me to get out of bed…The last few days, I couldn’t even bring myself to get out of bed.” (Gary, clinic).

Some adolescents were able to identify activities that they would and would not do. For example, some could put in enough effort to engage in passive activities, for example,

“So it’s kind of, putting in effort to go and do things that will decline. I would do more, kind of, passive things, so like TV and movies, where it’s just in front of you.” (Richard, community).

Other young people continued to take part in more demanding activities because they felt compelled or obligated.

**Theme 3—Losing a sense of connection and belonging:** “I’ll be there but I won’t be present” (Jennifer, clinic)

This theme focuses on adolescents’ connections with others and the world around them.

*Sub-theme: Feeling disconnected from others*

Young people described a sense of relatedness, where they knew other people had similar feelings or shared experiences, and that helped them to feel connected. Receiving direct social support was important, with adolescents describing the significance of having a “support network”. Receiving emotional support helped to improve their mood and motivation. In the absence of connection, adolescents often felt detached from others.

“Participant: “Yeah sometimes, like sometimes when I say no to my friends a lot they’ll just go and have a fun time and then they’ll be talking about it.

Researcher: How does that make you feel?

Participant: A bit, quite more lonely.” (Alice, clinic).

Many participants found it difficult to communicate or express their feelings to others and instead kept things internalised. Some felt an internal struggle; they wanted to talk about their feelings, but did not want to be a burden to others. Putting feelings into words was especially hard for those in the community who did not routinely discuss their emotions with others. Some masked their real feelings by pretending they were happy or enjoying experiences when they were not.

“I guess, most of the time things - I probably appear as though it excites me, but then inside I'm just like going along with everyone else. Like if they find it exciting, I will just be like 'yeah that's nice,' but I’ll probably find it really boring.” (Isla, community).

*Sub-theme: Feeling detached from the present reality*

As well as social connections, some young people experienced a disconnection from their surroundings, and/or a disconnection from themselves. This sub-theme featured more strongly in the community sample. When describing feeling disconnected from the moment, adolescents used phrases such as, “going through the motions” or being on “autopilot”. Some adolescents described this as if they were watching things happen from afar, like in a film or without any depth. One young person described this feeling as an out-of-body experience, as if watching themselves from above.

“I just go through the normal stuff, but being more looking on than actually doing it, it's more like it's looking through a film, and just my body doing exactly what it would have done anyway, with me in my head watching somehow, rather than me just being there.” (Tylor, community).

For many young people disconnecting from the world around them was also a deliberate distraction from their feelings or situations.

**Theme 4: Questioning sense of self, purpose, and the bigger picture:** “What’s the point in trying anymore?” (Maddie, community)

This theme reflects adolescents’ search for meaning and understanding, and their perception and beliefs about the world.

*Sub-theme: Reflecting on feelings, identity and purpose*

Adolescents described a loss of purpose, questioning the meaning of life and of taking part in day to day activities. This description was closely linked to not wanting to do things. A loss of purpose was described by adolescents in both the clinical and community samples.

“When I think in the more wide sense I realise that there's really no point to any of this, GCSEs, exams all of that, eventually we're all gonna die, what use does it really have.” (Stuart, community).

In contrast to lacking purpose, feeling the need to have a meaningful life, and thinking “I won’t have anything to look back on” (Isla, community) was the driving force for some young people to change their actions.

Young people differed in their ability or interest in self-reflection. A lot of adolescents expressed uncertainty, as in feeling like “I don’t know my feelings”. Some young people were in the process of discovering their identity, saying “I just kinda didn't realise my interest” (Ivy, clinic) and “[I’m] still trying to figure out what I like more” (Mel, community). Others displayed explicit insight into their feelings, and expressed this at a deeper level, often appearing self-critical and considering what their feelings said about them. Matthew (community) said “I thought I was a bit better than that, but clearly wasn’t.”.

*Sub-theme: Experiencing a lack of agency and a narrowing of perspective*

As well as searching for self-discovery, young people talked about their view of the wider world and often expressed a bleak outlook and a lack of personal agency. The majority of young people described feeling “stuck”, “trapped” or “enclosed”. This sometimes resulted in a “mental battle” between how they felt, i.e. no emotion, and how they wanted to feel, i.e. excited. At other times, this was experienced as “acceptance” and resignation.

“Like you don’t feel yourself. People point it out to you, and you don’t change ‘cos that’s how you feel.” (Amy, community).

When struggling with their emotions, a number of young people described a narrowing of their perspective. Some young people felt “there’s kind of no way of getting back to the way I was” (Joanne, community) and could not see beyond their current circumstances or emotional state. This was closely linked with adolescents having a bleak outlook on the future, having “a lack of overall optimism” (Neil, community), and not wanting to think long term, or believing that nothing would change.

### Connection between themes

**Fig. 2:**
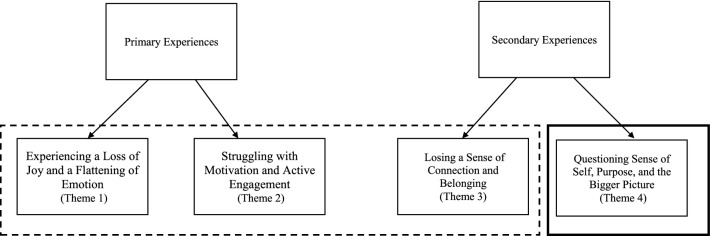
Diagram showing the relationship between themes. The primary experiencing problems were captured in themes one and two. The secondary experiencing problems were captured in themes three and four. A dotted line represents the themes which capture the emotional and behavioural components of adolescents’ experiences. A bold solid line represents the theme which captures the cognitions and interpretation of experiences

The salience of, and connection between, themes was considered. Themes 1 and 2 encapsulated the most prominent and central components of anhedonia, and Themes 3 and 4 incorporated secondary experiences related to, or part of anhedonia. In addition, Themes 1–3 captured the feelings and behaviours that comprised adolescents’ experiences and theme 4 identified the cognitions and interpretation of feelings and behaviours (see Fig. 2).

## Discussion

This study aimed to understand the experience of anhedonia in the context of adolescent depression. Although it is considered to be a core symptom of depression, the subjective experience of anhedonia in adolescents has only been investigated using self-report scales, and, therefore, little is known about its nature, and how it is experienced and described by adolescents. Our results indicate that young people have a variety of different experiences which form the symptom of anhedonia. This included a loss of positive affect, a blunting of all emotion, a loss of drive and willingness to exert effort, social withdrawal, dissociation, loss of purpose, and hopelessness. These experiences were reported by adolescents with a formal diagnosis of depression (clinical sample) and by those with elevated symptoms of depression (community sample). Overall, there was substantial overlap between the sub-samples and the analysis did not detect any meaningful differences. The experiences of the clinical group were more homogenous, which is likely due to the screening process for attending the clinic. No clear gender differences emerged from the data, but this is an interesting area for future exploration. The descriptions of anhedonia in this study challenge the framing of anhedonia as simply the loss of interest and pleasure [1].

The range of experiences captured as part of anhedonia share features with general accounts of depression, i.e. a bleak view of everything and isolation and cutting off from the world [25]. They also bear a close resemblance to the description of reward deficits that are characteristic of other disorders [38]. For example, the negative symptoms of schizophrenia include loss of motivation, emotional blunting, and social withdrawal, as well as the loss of interest and pleasure (particularly anticipatory pleasure) [1]. The results of this study highlight marked similarities between the accounts of anhedonia by young people with depression and depression symptoms and the narratives of young people who have schizophrenia [39]. Both groups report reduced motivation, enthusiasm, blunted affect, social withdrawal and lack of agency. There are also marked overlaps between the experiences of anhedonia described here and, the description of ‘apathy’ in Parkinson’s disease which is described as a lack of interest, enthusiasm or motivation [40]. No participants in the clinical group reported any psychotic symptoms, therefore, it is unlikely that these individuals were presenting with schizophrenia. Psychotic symptoms were not assessed in the community sample, so it is possible that these participants were experiencing prodromal depressive symptoms and would develop schizophrenia. However, prodromal symptoms often lack specificity, i.e. marked social isolation or withdrawal [41] and clinically these features could be indicative of a range of psychopathology, not just schizophrenia [42]. Due to the similarity of experiences across disorders, these findings suggest that anhedonia may be best understood by taking a trans-diagnostic approach, looking across disorders. The NIH Research Domain Criteria approach aims to classify mental disorders based on dimensions of observable behaviour, rather than clustering symptoms into disorders [43]. The similarity in the experience of reward-related deficits across disorders suggests that taking a more trans-diagnostic approach may be useful for assessing and treating anhedonia [44].

The data from this study provide some insights into the extent to which consummatory, anticipatory and motivational anhedonia can be distinguished by adolescents. Aspects of anhedonia were typically mentioned as co-occurring, but could often be distinguished from each other, for example, adolescents frequently described not *wanting* to do something, but if they did do it, they *enjoyed* it. In contrast, adolescents did not make a clear distinction between lack of anticipation (i.e. looking forward to experiences, feeling excited) and lack of enjoyment (i.e. feeling that something was fun or satisfying) and these experiences tended to be described as one. This may explain why most questionnaire measures of anhedonia have not been able to identify independent factors reflecting the subjective experience of anticipatory and consummatory pleasure (DARS [23]; ACIPS [21]), despite the fact that these components can be disambiguated at a neural level [13]. Our data suggest that it may not be possible to meaningfully distinguish between consummatory and anticipatory anhedonia via self-report measures in adolescents. Thus, the clinical assessment of anhedonia in adolescents may benefit from developing new non-verbal methods of assessing this construct.

We found that adolescents often struggled to imagine pleasurable events in the near future. This may reflect difficulties or deficits in anticipatory pleasure but may also reflect prospective memory problems. Typically, participants’ long-term goals and ambitions, e.g. going to university, were intact even though the young person reported feeling hopeless and said they did not look forward to experiences. This distinction between the negative near future and the more positive distant future indicates that when depressed young people maintain some positive aspirations. These may form a basis for psychological treatments that target and enhance positive mental imagery [45].

A number of questions about adolescents’ subjective experience of anhedonia remain unanswered. It was often hard for young people to distinguish between loss of positive affect and the presence of negative affect, e.g. feeling bored and feeling sad. Therefore, it is unclear if some experiences (i.e. feeling disconnected) are best represented as part of anhedonia, or if they reflect broader negative emotions associated with depression. A number of adolescents described a blunting of all emotion (positive and negative), which is consistent with evidence that young people with depression symptoms have blunted neural responses to both positive and negative stimuli [13].

### Strengths and limitations

It is a strength of this study that both adolescents with a diagnosis of depression and those who reported symptoms of depression but did not have a diagnosis were recruited. This provided some clinical diversity. However, no adolescents were recruited from in-patient units or complex services; and, therefore, it is possible that the most severe instances of anhedonia were not captured in this study. Likewise, there was some diversity among participants regarding socio-economic status (using free school meals as a proxy). However, the sample was not diverse in other aspects, for example geography and ethnicity. An aim of qualitative research is to understand the experiences of a specific sub-group, but it is of value to build on the findings of one study by conducting further studies with different samples.

This qualitative study provides rich data but the study is not designed to provide results that can be generalised to the broader populations of adolescents with depression. Future quantitative research would be needed to establish the extent to which the experiences described by the participants in this study reflect those of the broader population of adolescents with symptoms of depression.

## Conclusions

This study highlights the subjective experience of anhedonia in adolescent depression. Young people’s accounts revealed a wide range of challenges beyond loss of interest and pleasure, i.e. loss of motivation, sense of connection and trying to make sense of these experiences. Our data suggest that the current concept of anhedonia in depression captures a limited aspect of the experiences of young people and overlaps with the negative symptoms of schizophrenia. Young people found it difficult to identify different components of anhedonia, such as anticipatory and consummatory aspects. Thus, the clinical assessment of anhedonia in adolescents may benefit from developing new non-verbal methods of assessing this construct. Of particular interest and importance to assessment and treatment was that although young people reported elevated depression symptoms and many had a formal diagnosis, most reported that their long-term goals and aspirations were intact, even in the context of current feelings of hopelessness and low motivation.

## Electronic supplementary material

Below is the link to the electronic supplementary material.

Supplementary file1 (DOCX 16 kb)
