# Supplementary material for: Understanding anhedonia: a qualitative study exploring loss of interest and pleasure in adolescent depression
Source: Eur Child Adolesc Psychiatry. 2019 Jul 3;29(4):489–99. doi: 10.1007/s00787-019-01364-y (PMC7103575; doi:10.1007/s00787-019-01364-y)
Supplement: Supplementary file 1 — Supplementary file1 (DOCX 16 kb) [file 787_2019_1364_MOESM1_ESM.docx]

**SUPPLEMENTARY MATERIAL**

*Description of Qualitative Themes, Sub-themes, Codes, and Concepts within each Code*

| **Sub-theme** | **Overall Codes** | **Concepts within each code** |
| --- | --- | --- |
| **THEME 1. EXPERIENCING A LOSS OF JOY AND A FLATTENING OF EMOTION** | | |
| **SUB-THEME:**  **Feeling less positive emotion and experiencing a cycle of boredom** | Experiencing less enjoyment, happiness or contentment | Presence or absence of feelings relating to: enjoyment, fun, contentment, joy, happiness, satisfaction, sense of achievement, feeling good, or liking. |
|  | Experiencing a constant loop of boredom or monotony | Presence or absence of feelings relating to: interest, fascination, curiosity, variation, or intrigue. Or conversely boredom. |
|  | Not getting a buzz or thrill out of life | Presence or absence of feelings relating to: excitement, eagerness, can’t wait, anticipation, enthusiasm, exhilaration, thrill, or liveliness. This relates to current emotional states, as well as the expectation, and imagination of future events. |
|  | Feeling a depressing sadness or disappointment | Presence of absence of sadness (i.e. unhappiness, misery, sadness, crying, or feeling down) or disappointment (i.e. feeling let down, or despair). |
|  | Struggling with frustration, shame or self-doubt | Presence or absence of irritability, anger, dislike, hatred, jealousy or envy, or shame (i.e. embarrassment, shame, or self-conscious emotion). |
|  | Battling with worry, stress or academic pressure | Presence or absence of anxiety (i.e. worry, scared, anxiety, stress, pressure) |
| **SUB-THEME:**  **Feeling dampened emotions** | Experiencing a dulling down of emotions | Partial or complete loss or lack of emotion (either lack of positive, or neither positive or negative). |
|  | Experiencing complete blankness or indifference | A general flatness (i.e. dull, grey), blankness (i.e. feeling nothing) or indifference (i.e. not caring about anything). |
|  | Being overwhelmed by intense, repressed emotions | Feeling strong or an overwhelming amount and/or intensity of emotion. A sense of catharsis and feeling of relief from releasing strong or repressed emotions. |
|  | Fluctuating between strong highs and lows | Feeling or experiencing extreme highs and lows or sudden changes in emotions from one to another. |
| **THEME 2. STRUGGLING WITH MOTIVATION AND ACTIVE ENGAGEMENT** | | |
| **SUB-THEME: Feeling unmotivated but maintaining long term aspirations** | No longer wanting to do anything or reach goals | Presence or absence of drive, wanting to do things, feeling motivated, and being goal orientated. |
|  | Maintaining long term aspirations | Presence or absence of ambition and long term goals and aspirations. |
| **SUB-THEME: Feeling unable to gain momentum or engage in effortful activities** | Struggling to push forwards and gain momentum | Changes in effort exerted. Making more of an effort, feeling like things are more effort to do, pushing through or being pushed to do things, and trying/being invested in reaching a goal – doing things in order to get the end result. Or conversely making less effort, or not trying. |
|  | Engaging in passive but not effortful activities | Presence or absence of physical action or taking part in activities/experiences. Or making a distinction between passive and active activities i.e. only engaging in passive activities (i.e. watching TV) rather than active activities. |
| **THEME 3. LOSING A SENSE OF CONNECTION AND BELONGING** | | |
| **SUB-THEME: Feeling disconnected from others** | Creating a distance between oneself and others | Presence or absence of connection with other people, a sense of relatability, being similar to others or sharing experiences. Or conversely shutting off (physically and emotionally) and creating a barrier between themselves and others (i.e. social withdrawal). |
|  | Feeling isolated and distant from other people | Presence or absence of social support from others, feeling understood, listened to, and helped. Or conversely feeling or being left to handle things without help or being called an attention seeker. |
|  | Internalising or masking real emotions | Presence or absence of communication with others, talking about/expressing emotions to others. Or conversely keeping feelings inside (i.e. internalising), or masking real feelings and/or pretending to other people. |
| **SUB-THEME: Feeling detached from the present reality** | Feeling disconnected from the present moment | Feeling disconnected from the moment/reality, watching things happen from afar, like through a film/ without depth. Or feeling a sense of disconnection and unreality in one’s personal self, like watching self/ outer body experience. |
|  | Distancing oneself from the real world | Escaping into another world/ reality (i.e. computer game) or distracting self from current situation by doing something else. Could be used as a coping strategy. |
| **THEME 4. QUESTIONING SENSE OF SELF, PURPOSE, AND THE BIGGER PICTURE** | | |
| **SUB-THEME:**  **Reflecting on feelings, identity and purpose** | Losing a sense of purpose | Importance or loss of purpose, motive/reason, meaning, not seeing the point, including questioning existence. |
|  | Reflecting on experiences and how they view themselves | Presence or absence of self-awareness, insight about themselves, reflection on how they feel about or view themselves. Considering self-image, identity and view of the self, as well as self-criticism, self-confidence and self-esteem. Or conversely, not considering the impact of their experiences on how they view themselves. |
|  | Figuring out experiences or living with uncertainty | Figuring out and making sense of experiences, situations or emotions i.e. what they like, don’t like. Or conversely experiencing uncertainty, unsure of what they are feeling or experiencing. Or simply accepting their experiences without trying to understand or make sense of their feelings or experiences. |
| **SUB-THEME: Experiencing a lack of agency and a narrowing of perspective** | Losing a sense of personal agency | The presence or absence of personal agency. Feeling/ believing they do or do not have the power or ability to control how they feel. Resigning themselves to the fact there is nothing they can do to change their emotions. Feeling restricted or guided by social institutions (i.e. school) and societal norms (i.e. parenting). |
|  | Being unable to see the bigger picture | A shift or change in perspective, viewing things differently i.e. not seeing the bigger picture, or beyond the current problem or situation. Also feelings of hopelessness, and having a bleak outlook on things to come. Or conversely feeling hopeful and optimistic. |
| **Wider Context** | | |
|  | Broader Personal Circumstances | The description of unique personal circumstances (i.e. domestic abuse). |
|  | Past or current mental health support | The description of mental health support, which includes support from NHS services and school counselling or services (i.e. teacher involvement). This also includes expectations or changes in depression or anhedonia as a result of treatment. |
